# Supplementary material for: Conditional chemoconnectomics (cCCTomics) as a strategy for efficient and conditional targeting of chemical transmission
Source: eLife. 2024 Apr 30;12:RP91927. doi: 10.7554/eLife.91927 (PMC11060718; doi:10.7554/eLife.91927)
Supplement: Figure 3—source data 3. [file elife-91927-fig3-data3.zip › Figure 3 source data3 part 1.zip!/Figure 3-Source data 3-part1/1081-1-CRISPResso_Report_8zosk6yj.zip!/CRISPResso_on_1081_3/1a.Read_barplot.pdf]

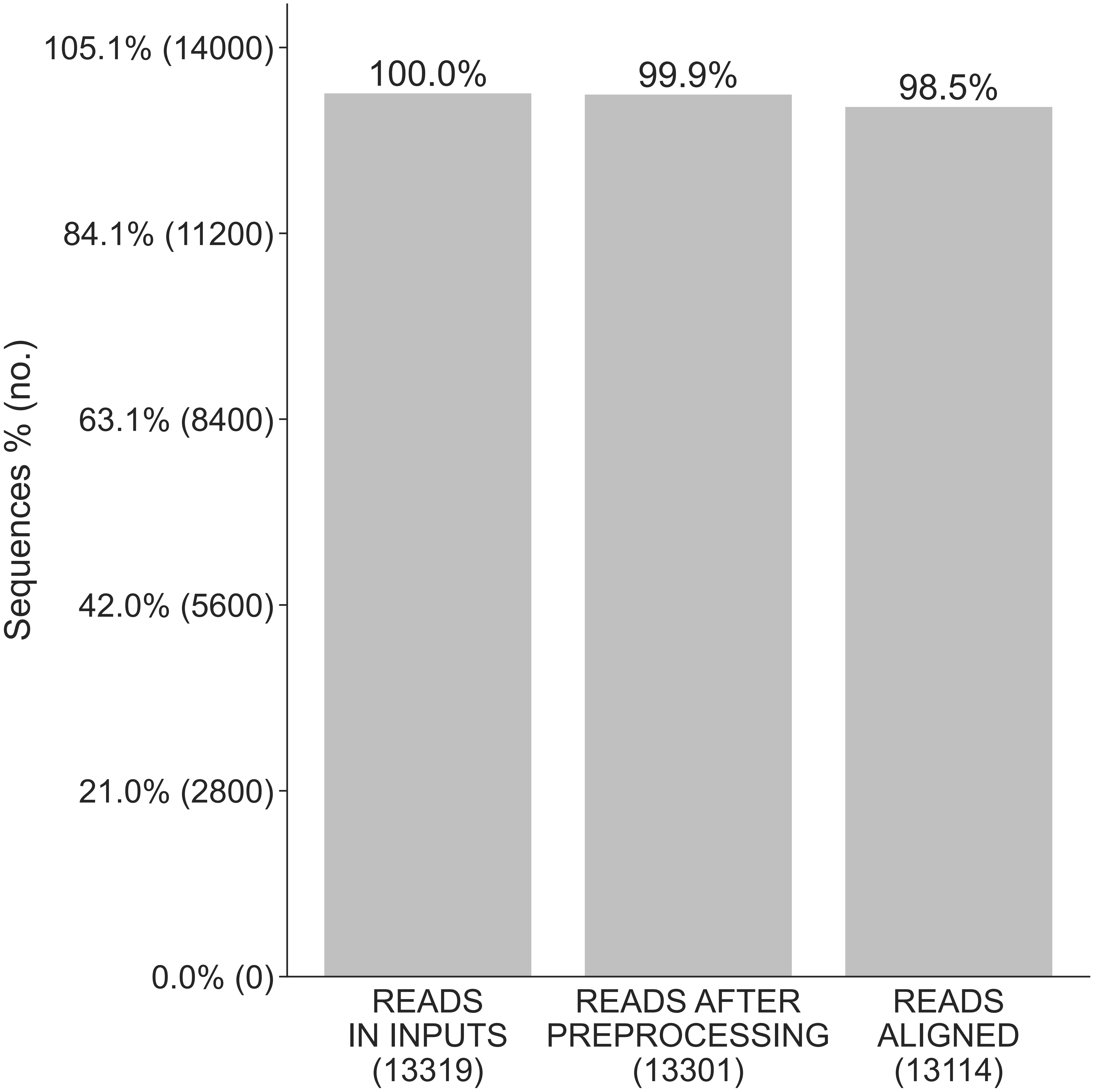

Sequences % (no.)
105.1% (14000)
84.1% (11200)
63.1% (8400)
42.0% (5600)
21.0% (2800)
0.0% (0)
100.0%
99.9%
98.5%
READS IN INPUTS (13319)
READS AFTER PREPROCESSING (13301)
READS ALIGNED (13114)
